# Effect of a GnRH analogue (peforelin) on the litter performance of gilts and sows

**DOI:** 10.1186/s40813-017-0054-5

**Published:** 2017-03-15

**Authors:** Ellen de Jong, Jan Jourquin, Johannes Kauffold, Steven Sarrazin, Jeroen Dewulf, Dominiek Maes

**Affiliations:** 1 Department of Reproduction Obstetrics and Herd Health, Unit Porcine Health Management, Faculty of Veterinary Medicine, University of Ghent, Salisburylaan 133, B-9820 Merelbeke, Belgium; 2 Elanco, S.A. Eli Lilly Benelux N.V., Stoofstraat 52, B-1000 Brussels, Belgium; 3 Large Animal Clinic for Theriogenology and Ambulatory Services, Faculty of Veterinary Medicine, University of Leipzig, An den Tierkliniken 19, 04103 Leipzig, Germany; 4 Department of Reproduction Obstetrics and Herd Health, Veterinary Epidemiology Unit, Faculty of Veterinary Medicine, University of Ghent, Salisburylaan 133, B-9820 Merelbeke, Belgium; 5 Present address: Flemish Animal Health Service (Dierengezondheidszorg Vlaanderen), Industrielaan 29, B-8820 Torhout, Belgium

**Keywords:** Sows, Gonadotropins, Peforelin, Prolificacy, Birth weight

## Abstract

**Background:**

Maintaining optimal reproductive and litter performance is essential for meeting economic targets in commercial pig production. Treatment with exogenous gonadotropins in sows after weaning or in gilts after altrenogest treatment has been used to stimulate follicular development leading to more piglets born and eventually higher birth weights. The effect of peforelin on litter performance was investigated in 212 gilts, primi- and pluriparous sows in three herds. Animals were randomly allocated to three treatments 24 h after weaning: peforelin (P group), eCG (E group), and physiological saline solution (C group). Numbers of total, liveborn and stillborn piglets and mortality rate during lactation were recorded. Birth weights and coefficient of variation in weights within litter were assessed. All parameters were compared among treatments.

**Results:**

Over all parities, no difference was found among treatments in litter size nor mortality rate, but birth weights were significantly lower in the E group. Stillbirth numbers in pluriparous sows were 2.2, 0.9 and 1.4 for P, E and C groups, respectively (*p* = 0.04). Piglets in the P group had significantly higher live born birth weights in gilts, compared to the E group (1.36, 1.26, 1.32 kg (*p* < 0.02) for P, E and C group, respectively). No significant differences were found for the other investigated parameters.

**Conclusions:**

Peforelin treatment showed no improvement of litter performance compared to no treatment.

## Background

Maintaining optimal reproductive and litter performance is essential for meeting economic targets in commercial pig production. Treatment with exogenous gonadotropins in sows after weaning or in gilts after altrenogest treatment has been used to stimulate follicular development [1–3]. Follicular stimulation could lead to a better quality of the oocytes and to better embryo viability [4–6], subsequently leading to a higher number of piglets born [7] and eventually higher birth weights [8].

The release of luteinizing hormone (LH) and, to a lesser extent, follicle-stimulating hormone (FSH) from the pituitary gland is governed by the hypothalamic gonadotropin-releasing hormone (GnRH) [1, 9–11]. GnRH is therefore a key regulator of the growth, maturation, and ultimately, the ovulation of follicles. While LH secretion is only dependent on GnRH, FSH is also regulated by other peptides, such as gonadal activins, inhibins and follistatins [11–13]. Twenty years ago, Sower et al. (1993) demonstrated for the first time that there is another selective FSH-releasing factor produced by the hypothalamus in fish, more specifically in the lamprey, *Petromyzon marinus* (lamprey GnRH-III, l-GnRH-III) [14]. Products, which have l-GnRH-III (peforelin) as the active substance, are marketed for the induction of estrus in sows after weaning and in sexually mature gilts after progestagen therapy. Different studies conducted in Germany and Belgium have confirmed that treatment with peforelin has positive effects on estrus induction in gilts and sows [15–17].

Peforelin could positively influence the oocyte quality, ovulation rate, embryonic survival and litter weight. This positive influence on embryonic survival and litter weight was suggested by Jourquin and Goossens (2011) and Vangroenweghe et al. (2013) in litters from peforelin treated sows [18, 19]. The mortality rate of litters born to peforelin treated sows was significantly lower (14 versus 17%) and the birth weight was significantly higher (average of 1.24 versus 1.20 kg) than in litters from untreated control sows. However, no comparison was made with another gonadotropin-like product and no data were available on the homogeneity of the litters.

The purpose of the study reported herein was to investigate the effect of peforelin on subsequent litter performance in gilts after altrenogest treatment and in post-weaning sows in Belgian sow herds. The performance of the peforelin treated animals was compared to that of a pregnant mare serum gonadotropin (eCG) treated group and a saline treated control group.

## Methods

The study was conducted between January 2010 and May 2011.

### Study animals and management practices

This study was part of a larger study, of which the main objective was to demonstrate differences in estrus rate between the treatment groups [17]. Statistical sample size calculations were performed in relation to this main objective. Of a total of 1945 gilts and sows housed in three sow herds, 262 breeding animals were randomly selected. In Table 1, more detailed information on the farms is presented. The animals were stratified in three age categories, *i.e.* 86 gilts, 87 primiparous and 89 pluriparous sows. Animals with clinical disease and/or reproductive disorders, such as puerperal disease or pathological vaginal discharge were not included.

**Table 1:** Characteristics of the three pig herds included in the study

	Herd A	Herd B	Herd C
Number of sows per herd	1200	1700	600
Number of sows included in study	56	87	119
Breed of sows	Danbred x York	PIC	Topigs20
Batch-production-system for sows (weeks)	1	2	4
Lactation period (weeks)	3	3	3
Piglets weaned/sow/year	25.9	26.1	26.3
Age of gilts at first insemination (days)	280	290	250

Estrus stimulation started on the first day post weaning (pw) in sows or 48 h after the last altrenogest treatment in gilts (for the sake of convenience and consistency, the day of the last altrenogest treatment is henceforth referred to as the first day post-weaning or ‘pw’), using at least two teaser boars. All animals were fed *ad libitum* with a gestation feed from day one pw until insemination. A supplement of 150 mg dextrose per day per animal was provided as a top dressing. To further optimize estrus stimulation and detection, supplemental boar noises were played to the animals in herd A via a voice recorder, and herd C used a Contact-O-Max (Ro-Main Europe, France), which is a remote controlled mobile unit with a boar inside.

Estrus detection was performed twice a day (am and pm) from day four pw onwards. The same artificial insemination (AI) schedule was used in all three herds. Briefly, sows showing standing estrus on day 4 pw in the morning were inseminated 24 h later, and those showing estrus in the evening were inseminated 12 h later. Sows showing standing estrus on day five were inseminated 8 h later, while those showing estrus on day 6 pw were inseminated immediately. Sows that still showed estrus 12 h after their first round of AI were inseminated a second time, and a third time in the rare cases where standing estrus persisted for 24 h. Single sire semen from boars of proven fertility was purchased from a commercial AI centre.

Pregnancy testing was performed by the herd veterinarian using trans-abdominal ultrasound scans performed with a sectorial probe at 23 to 28 days after insemination and again two weeks later. Gilts and sows that were found to be pregnant at day 23 to 28 were moved to the gestation unit. In herds A and B, pregnant females were housed in groups, with the exception of gilts and sows that had previously experienced reproductive problems (*e.g.* repeat breeding) in herd A. In herd C, only pregnant gilts were housed in groups, and pregnant sows were housed in individual stalls as was still in accordance with EU legislation in 2010. In all three herds, animals were fed *ad lib* a gestation diet after confirmed pregnancy. Sows were moved to the farrowing unit approximately one week before the expected farrowing date.

To obtain equal litter sizes (12–13 piglets/litter), cross fostering of piglets was allowed within 24 h after farrowing, but only among sows of the same treatment group and after first weighing (<12 h after birth). Therefore, piglets were individually identified at first weighing using ear tags with different colors according to the treatment. Piglets in all three herds were weaned after twenty to twenty-two days of lactation.

### Experimental design

Within each herd and each age category, animals were randomly allocated to one of three treatment groups prior to treatment (Tables 2, 3 and 4): 1) peforelin (the P group), in which gilts and pluriparous sows were treated with 150 μg peforelin (2 ml Maprelin®, Veyx-Pharma, Schwarzenborn, Germany) based on the manufacturers’ instruction, and primiparous sows with 37.5 μg peforelin (0.5 ml Maprelin®) [20]; 2) equine Chorion Gonadotropin (eCG; the E group) as a positive control, in which animals were treated with 1000 IU eCG (1 ml Folligon®, MSD Animal Health, Brussels, Belgium) and 3) physiological saline solution as a negative control (the C group), in which animals were treated with 1 ml of 0.9% NaCl sterile solution (Baxter, Lessen, Belgium). All treatments were applied via intramuscular injection into the neck 24 (±1) h post weaning (sows) or 48 (±1) h after the last altrenogest administration (gilts). The entire study, including AI and the recording of the different parameters, as described below, was conducted using a blinded design.

**Table 2:** Mean back fat levels at the different time points in the different parity and treatment groups (with P = peforelin, E = eCG, C = control and BF1 = back fat measured one month after insemination; BF2 = back fat measured at farrowing; BF3 = back fat measured at weaning; BF3-BF2 = calculated back fat loss during lactation and SD = standard deviation) in 212 sows in three herds

	Group	n	BF_1_ ± SD (mm)	BF_2_ ± SD (mm)	BF_3_ ± SD (mm)	BF_3_-BF_2_ ± SD (mm)
Gilts	P	28	16.5 ± 3.5	21.0 ± 4.2	17.5 ± 4.2	−3.3 ± 2.6
	E	17	17.7 ± 2.1	22.9 ± 3.5	18.9 ± 2.8	−3.6 ± 3.2
	C	25	17.9 ± 2.5	22.3 ± 3.8	19.1 ± 3.7	−3.4 ± 3.4
Primiparous	P	30	19.0 ± 3.3	21.9 ± 5.6	18.6 ± 4.5	−4.6 ± 3.3
	E	15	16.5 ± 3.4	20.7 ± 3.1	16.5 ± 3.9	−4.2 ± 2.1
	C	24	17.6 ± 3.4	20.6 ± 3.3	16.7 ± 3.4	−3.4 ± 3.3
Pluriparous	P	20	16.2 ± 4.1^b^	21.2 ± 6.1	16.4 ± 3.2	−4.0 ± 2.9
	E	27	19.7 ± 4.6^a^	24.1 ± 4.7	19.1 ± 4.5	−3.3 ± 4.2
	C	26	17.8 ± 3.8^a,b^	21.0 ± 4.2	17.8 ± 3.6	−4.3 ± 3.6
All parities	P	78	18.4 ± 3.6	22.2 ± 4.5	18.1 ± 4.0	−4.0 ± 3.0
	E	59	17.5 ± 3.5	21.8 ± 4.3	18.3 ± 4.0	−3.4 ± 3.7
	C	75	17.2 ± 3.6	21.3 ± 4.8	17.4 ± 3.7	−4.0 ± 3.1

^a, b:^ Within a specific parity group, differences among treatment groups were statistically significant (*p* ≤ 0.05)

**Table 3:** Number of total born (TB), live born (LB) and stillborn (SB) piglets according to treatment (P = peforelin, E = eCG, C = control) and parity of 212 litters of three herds

	Group	n	Litter numbers (mean ± standard deviation)		
			TB	LB	SB
Gilts	P	28	13.1 ± 3.4	12.3 ± 2.9	0.7 ± 0.9
	E	17	15.2 ± 2.8	13.5 ± 3.1	1.6 ± 2.7
	C	25	14.2 ± 3.5	13.6 ± 3.4	0.6 ± 1.0
Primiparous	P	30	14.8 ± 3.9	13.6 ± 3.4	1.0 ± 1.8
	E	15	15.7 ± 3.2	14.4 ± 2.9	1.3 ± 1.5
	C	24	14.0 ± 3.0	13.3 ± 3.1	0.5 ± 0.9
Pluriparous	P	20	15.1 ± 3.7	12.4 ± 3.8	2.2^a^ ± 0.5
	E	27	14.9 ± 4.4	13.7 ± 3.8	0.9^b^ ± 1.0
	C	26	14.4 ± 4.4	12.9 ± 4.2	1.4^a,b^ ± 2.3
All parities	P	78	14.3 ± 3.7	12.8 ± 3.3	1.3 ± 1.8
	E	59	15.2 ± 3.7	13.8 ± 3.4	1.2 ± 1.8
	C	75	14.2 ± 3.6	13.2 ± 3.6	0.9 ± 1.6

^a, b^ Within a specific parity group, differences among treatment groups were statistically significant (*p* ≤ 0.05)

**Table 4:** Mean birth weight ± standard deviation (BW ± SD, in kg) and coefficient of variation (CV) based on the sum of live born and stillborn piglets (LB + SB) and based on LB piglets only, and mortality rate (MR) according to treatment (*P* = peforelin, E = eCG, C = control) and parity of 3014 piglets (2688 LB) in 212 litters in three herds

Parity	Group	n	Weight				MR (%)
			BW_LB + SB		BW_LB		
			Mean ± SD	CV	Mean ± SD	CV	
Gilts	P	28	1.33^a^ ± 0.37	0.19	1.36^a^ ± 0.34	0.22	9
	E	17	1.23^b^ ± 0.31	0.22	1.26^b^ ± 0.30	0.22	13
	C	25	1.29^a,b^ ± 0.34	0.21	1.32^a,b^ ± 0.32	0.21	12
Primiparous	P	30	1.42 ± 0.41	0.25	1.47 ± 0.39	0.22	12
	E	15	1.37 ± 0.36	0.27	1.40 ± 0.35	0.25	13
	C	24	1.42 ± 0.40	0.25	1.45 ± 0.37	0.23	12
Pluriparous	P	20	1.36 ± 0.43	0.25	1.41 ± 0.40	0.26	15
	E	27	1.34 ± 0.39	0.26	1.38 ± 0.36	0.23	16
	C	26	1.38 ± 0.48	0.25	1.43 ± 0.46	0.23	12
All parities	P	78	1.37^a^ ± 0.40	0.22	1.42^a^ ± 0.38	0.23	12
	E	59	1.31^b^ ± 0.36	0.25	1.35^b^ ± 0.35	0.23	14
	C	75	1.36^a^ ± 0.41	0.23	1.40^a^ ± 0.39	0.22	12

^a, b^ Within a specific parity group, differences among treatment groups were statistically significant (*p* ≤ 0.05)

### Data recording and calculated measures

From each litter, the number of total born, live born, stillborn and mummified piglets was noted. All piglets (live and stillborn) were individually identified and weighed within 12 h after birth, but before cross fostering. The coefficient of variation was calculated as the standard deviation divided by the mean, to assess the weight variations within a litter. Mortality rate was used to describe pre-weaning mortality.

Back fat levels of gilts and sows were measured one month after AI following treatment (BF1), the day of farrowing (BF2) and of weaning (BF3). The measurements were performed at the P2 position [21] by one operator using ultrasonography (linear probe, Tringa, Pie Medical ESAOTE, Belgium, The Netherlands and Luxemburg). Differences among BF3 and BF2 were calculated in order to determine the losses in back fat during lactation.

### Statistical analysis

Of the initial 262 animals, sixteen gilts and thirty-four sows were not pregnant or had incomplete records and were excluded from the analysis. Results for a total of 212 animals were included in the statistical analysis: 70 gilts, 69 primi- and 73 pluriparous sows. Statistical analysis was performed using version 20.0 of the SPSS software package (SPSS Inc., Chicago, Illinois, USA). Normal distribution of the data was tested using the Kolmogorov-Smirnov-test and the Shapiro-Wilk-test. The results for the different treatment groups were expressed as arithmetic means and the corresponding standard deviations (SD). For each outcome of interest a linear model was fitted (General Linear Model procedure in SPSS) and results were compared among treatment groups and among treatment groups and age categories by using the *post hoc* Scheffé test to adjust the *p*-values for the multiple comparisons. The analyses for outcome variables at the sow level (litter numbers and mortality rate) were adjusted for back fat levels whenever back fat was significantly associated with the outcome (*p* < 0.05). Furthermore, herd was included in the analyses as random effect to account for clustering of piglets and sows within herds. A significance level of *p* ≤ 0.05 was employed.

## Results

The only significant difference for back fat measurements was found in the pluriparous sows one month after insemination (BF1), with the sows in the P group having the lowest back fat (16.2 ± 4.1 mm) compared to the sows in the E group (19.7 ± 4.6 mm, *p* = 0.01). No significant differences were found among any of the treatments for the other parity groups at any time for the back fat measurements, nor for the calculated back fat losses during lactation (Table 2).

Table 3 shows the numbers of total born, live born and stillborn piglets according to parity and treatment. Litter size was numerically greater in the E group in all parity groups except for the pluriparous sows. Only the number of stillborn piglets in pluriparous sows was significantly higher in the P group, compared to the E group (*p* = 0.04), but not compared to the C group (*p* > 0.05). The number of mummified piglets per litter over all parities was similar for all treatment groups: 0.2 ± 0.6, 0.2 ± 0.5 and 0.1 ± 0.4 mummies for the P, E and C group respectively (*p* > 0.05).

In total, 3014 piglets (2688 live born) were individually weighed at birth (Table 4). The average birth weight both with and without stillborn numbers, was significantly higher in the P group, compared to the E group (*p* ≤ 0.02), and numerically higher, compared to the C group in gilts. On average a higher birth weight was observed in the P and C group compared to the E group over all parity groups both for total born piglets and for live born piglets alone (*p* < 0.01; Table 4).

No significant differences among any of the treatments for each parity group were found with respect to mortality rate during lactation (Table 4).

## Discussion

This study investigated the effects of peforelin, *i.e.* synthetic l-GnRH-III, on the litter performance of gilts after altrenogest treatment and of post-weaning sows in commercial Belgian pig herds, compared to treatment with eCG and no treatment. As this study was part of a larger trial, of which the main objective was to demonstrate differences in estrus rate between the treatment groups [17], statistical sample size calculations were performed in relation to this main objective. Therefore the power of the statistical analysis in the current study is often quite low, possibly resulting in non-significant differences. However, even with low power some significant differences could yet be demonstrated.

No significant differences were observed among the negative control group and the group treated with peforelin considering litter size and mortality rate. The birth weights of the piglets in the eCG group were lower in all parity groups and in gilts.

Although, litter size was numerically higher in gilts treated with eCG, compared to no treatment or peforelin treatment. The effect of treatment with supplemental LH-like activity products (such as eCG) was shown to be age dependent [22]. Therefore it is possible that the endogenous LH support of older sows is adequate enough to support follicular development, whereas that of gilts is maybe insufficient. Treatment with eCG in gilts and younger sows could thus have more influence on the outcome of total born piglets, compared to the litter size in older sows. The lack of significant differences with respect to litter size among the control and treatment groups in sows is consistent with the results of earlier studies [22, 23]. do Lago et al. (2005) and Martinat-Botté et al. (2010) found that eCG treatment increased the ovulation rate [2, 24], leading to larger litter sizes. This was the case for the eCG treated group in gilts in this study. Hence, the lower birth weights in this treatment group over all parity groups was possibly caused by the differences in total and live born piglets, as piglets born in a large litter mostly have lower birth weights, compared to piglets in small litters [25–28].

Several environmental factors, *e.g.* ventilation, nutrition, farrowing supervision may influence the stillbirth rate [29]. The overall management practices around farrowing were similar for all treatment groups within each of the three herds, therefore the higher stillborn number in the P group of the pluriparous sows was probably not caused by environmental factors. A lower birth weight was found to increase the probability of stillbirth [30] and, *vice versa*, a higher birth weight (>1.35 kg [31]) could lead to more birth difficulties, due to the relatively large size compared to the maternal pelvis, leading to asphyxia and likely to more stillborn piglets [32]. Although the birth weight of the stillborn piglets was significantly higher in the P group of the pluriparous sows, compared to the E group (data not shown), it did not exceed 1.35 kg, thus a higher number of stillborns due to difficulties during farrowing is doubtful. Sow factors, such as body condition and farrowing duration have also been shown to influence the number of stillborn piglets [29]. The back fat of the pluriparous sows at farrowing was similar for the P and C group and was approximately 20 mm, which would not have detrimental effects on the number of stillborns [21]. The farrowing duration was not measured in this trial, therefore no conclusion can be drawn on a possible influence of this parameter.

Previous studies have shown that l-GnRH-III treatment could increase FSH levels [13, 33–35]. Increased levels of FSH during the follicular phase increase follicular size [4, 36]. It was hypothesized that treatment with peforelin results in a more uniform pre-ovulatory pool, containing more competent and larger follicles to ovulate [20]. A more uniform pre-ovulatory follicle pool may result in a more uniform oocyte quality [37] and more uniformly developed embryos [38, 39], which could finally result in more uniform birth weights [8, 18, 19]. It has been shown that animals treated with peforelin, similar as in the present study, had larger preovulatory follicles than control and eCG treated animals [17, 20]. The coefficient of variation of birth weights is a measure of the homogeneity of the piglets’ weight at birth. A low within-litter-variation in birth weight is beneficial, as this is positively associated with survival and performance of the piglets [40, 41]. However, no significant differences were found in the coefficient of variation in none of the parity groups in this study.

## Conclusion

In conclusion, peforelin treatment showed no difference compared to no treatment based on litter performance. On the opposite, litter size seems to be numerically higher in gilts, treated to eCG, compared to control of peforelin treated groups, however, the differences between groups were not statistically significant. A possible consequence of the higher litter size, is the lower birth weight of the piglets.

## Abbreviations

AI: Artifical Insemination
BF: Back Fat
BW: Birth weight
eCG: Equine Chorion Gonadotropin
FSH: Follicle stimulating hormone
GnRH: Gonadotropin Releasing Hormone
LB: Live born
l-GnRH-III: Lamprey – Gonadotropin Releasing Hormone - III
LH: Luteinizing hormone
MR: Mortality rate
pw: post weaning
SB: Still born
SD: Standard deviation
TB: Total born
